# Many small steps towards a COVID-19 drug

**DOI:** 10.1038/s41467-020-18710-3

**Published:** 2020-10-07

**Authors:** Daniel A. Erlanson

**Affiliations:** Frontier Medicines Corporation, 151 Oyster Point Blvd., 2nd Floor, South San Francisco, CA 94080 USA

**Keywords:** Structural biology, Drug discovery

## Abstract

A large-scale screening campaign has yielded dozens of crystal structures of small molecule fragments that bind to the main protease of SARS-CoV-2. The global research community is encouraged to pursue these as drug discovery starting points for COVID-19.

As of July 2020, more than half a million people worldwide have died of COVID-19. That number will have grown considerably by the time you read this. The research community has responded to the pandemic with unprecedented urgency. Multiple experimental vaccines have entered clinical trials—with several already in phase III—while clinicians explore potential treatments using experimental antibodies and drugs already approved for other indications. With luck, an effective vaccine will soon be available. However, it is worth remembering that while we still do not have an effective vaccine for HIV nearly three decades after its discovery, small molecule drugs have made living with the virus manageable. Creating new small molecule drugs often takes years, which is all the more reason to start early. Some promising steps towards the goal of developing a drug that specifically targets SARS-CoV-2, the coronavirus responsible for COVID-19, are now described by Douangamath et al. in *Nature Communications*^1^. Crucially, all the information reported in the manuscript was released before publication, with the idea that the wider scientific community can rapidly build upon it.

## A large-scale fragment screen against the SARS-CoV-2 main protease M^pro^

SARS-CoV-2 enters human cells and coopts ribosomes to translate its viral RNA into two polyproteins. These polyproteins are in turn cleaved into individual peptides, largely by an enzyme prosaically called the main protease, or M^pro^. Because of its early, essential role in the viral replication cycle, M^pro^ is an obvious target for drug discovery. Indeed, researchers have previously identified potent peptidomimetic inhibitors of M^pro^ from MERS-CoV, a related coronavirus^2^. Based on this work, inhibitors of SARS-CoV-2 M^pro^ have also been rapidly developed^3,4^, but their peptidic nature may complicate oral delivery.

In order to identify new, non-peptidic leads, an international team led by Martin Walsh and Frank von Delft from Diamond Light Source, UK and Nir London from the Weizmann Institute of Science in Israel tackled M^pro^ using an approach called fragment-based drug discovery (FBDD)^5^. Rather than starting from a larger, substrate-based molecule as with the peptidomimetics, or screening hundreds of thousands of drug-sized molecules, FBDD starts with more limited libraries of smaller molecules, or fragments. Because there are fewer possible small fragments than drug-sized molecules, FBDD can survey chemical space more comprehensively to find the most attractive starting points for medicinal chemistry. Also, because fragments are so small, they tend to bind to more sites on proteins, which facilitates lead identification. Nearly 50 FBDD-derived drugs have entered clinical development.

The active site of M^pro^ contains a cysteine residue essential for catalytic activity, and the previously reported SARS-CoV-2 M^pro^ inhibitors contain an electrophilic center (such as an aldehyde or an α-ketoamide) that covalently traps the cysteine thiol^3,4^. Covalent bond formation can improve the efficacy of inhibitors, particularly when the bond formation is irreversible, as is the case for drugs such as penicillin. Starting with the era of high-throughput screening, however, pharmaceutical companies tended to move away from covalent drugs for fear that indiscriminate binding to other proteins could cause unpredictable toxicity. Yet the recent success of multiple selective, well-characterized covalent cancer drugs has renewed interest in covalent modifiers, including fragment-sized molecules^6,7^.

In the new *Nature Communications* paper, the researchers started by screening a library of ~1000 electrophilic fragments against M^pro^ using mass spectrometry to identify which ones could bind to the protein. Due to the reactivity of the active-site cysteine, rather stringent conditions (5 µM of each fragment for 1.5 hours at room temperature) were used to achieve some degree of discrimination. Under these conditions, 68 fragments appreciably modified M^pro^ but were not generically reactive against other proteins or small molecule thiols.

Among the various methods used to characterize fragments that bind to a protein, X-ray crystallography is arguably the most informative as it reveals the detailed molecular interactions of ligand binding. Even when successful, crystallography can be time-consuming, and many drug hunters consider themselves fortunate when they have one or a few crystal structures. Here the researchers obtained a 1.25 Å high-resolution structure of M^pro^ from crystals suitable for soaking experiments. Some of the covalent fragments were first reacted with the protein and then crystallized. In total 68 covalent fragments and 1176 mostly noncovalent fragments were screened by either co-crystallization or soaking of the compounds into the crystals. 1877 crystals were mounted at Diamond and 1638 datasets with a resolution better than 2.8 Å were collected—an impressive undertaking that yielded structures of 96 fragments bound to M^pro^. The speed with which this was done is breathtaking: protein crystals were first obtained on 13 February 2020, and all experimental data were collected by 7 March. The first crystal structures were made public only three days later, and by the beginning of April 2020 all final structures were released in the midst of the evolving pandemic. If a picture is worth a thousand words, Douangamath et al. have published a novel.

**Fig. 1:**
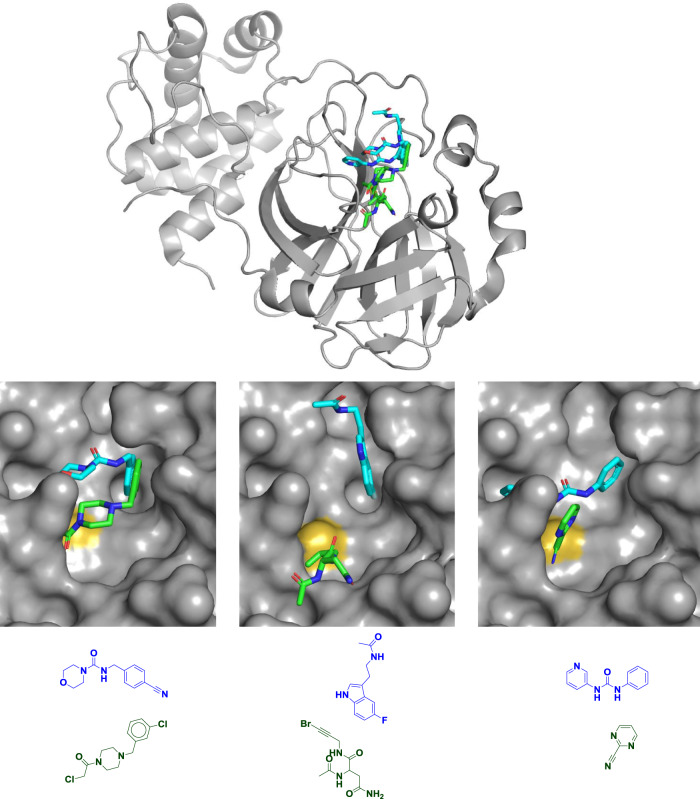
Diverse fragments in diverse binding sites. M^pro^ structure and chemical structures of six fragments bound to the active site of M^pro^ to illustrate their multiple binding locations. Although the catalytically active enzyme is an obligate homodimer, only one monomer is shown (top) for clarity. Covalent fragments are shown in green, noncovalent fragments are shown in blue or cyan, and the catalytic cysteine sulfur atom is shown in yellow. All six fragments are shown overlaid in the top panel, in pairs in the middle panel, and as chemical structures in the bottom panel; overlays are shown to illustrate the range of binding modes though each fragment was crystallized individually. All three noncovalent fragments shown come from a “poised” fragment library designed for rapid follow-up chemistry through the urea or amide linkages^13^. Most of the covalent fragment hits disclosed are chloroacetamides (bottom left), but others include bromoalkynes (bottom middle) and aromatic nitriles (bottom right). PDB codes are (from left to right and top to bottom): 5RFE, 5R7Z, 5R83, 5RET, 5RG3, and 5RHB. As noted by Douangamath et al.^1^, overlaying structures suggests many opportunities for fragment merging, for example the two compounds on the left.

Not surprisingly most of the fragments—including all 48 covalent ones—bind in the active site. Interestingly, two fragments that bind covalently to the active-site cysteine were expected not to be covalent; the bromoalkyne “warhead” they contain (Fig. 1) is generally unreactive^8^. The fact that these compounds modify the enzyme illustrates how susceptible the active-site cysteine is to modification, which could auger well for drug discovery. That said, the multiple recently approved kinase inhibitors all target noncatalytic cysteines^7^ and few approved covalent drugs target active-site cysteine residues. That fact speaks to the importance of 23 non-covalent fragments which collectively explore many individual subpockets within the large active site. Indeed, recent fragment-based efforts against another cysteine hydrolase, USP7, led to two classes of potent, noncovalent inhibitors^9,10^.

Additionally, M^pro^ functions as a dimer, and three fragments bind at the dimer interface; the researchers suggest that these could be optimized into molecules that would allosterically inhibit the enzyme. Examples of some of the fragments illustrating the multiple binding modes and interactions are shown in Fig. 1.

Another impressive aspect of the work is the researchers’ dedication to open science. Coordinates for all the structures have been deposited in the Protein Data Bank and were released in real time as they were solved. The next steps are to use this information to produce molecules with better activities to seed drug discovery programs. These methods commonly include merging or linking two fragments to produce a larger, more potent molecule as for example in the case of cancer drug venetoclax^11^. The researchers highlight several possibilities, one of which is shown in Fig. 1. More commonly, fragments can be grown into larger molecules by adding new moieties at promising sites as was done for the cancer drug vemurafenib^12^. Some of the fragments disclosed were specifically designed to have functional groups well-suited for easy chemistry and thus facilitate this process^13^.

The protean nature of fragments provides ample opportunities for chemical elaboration, and a single fragment can lead to multiple drugs. For example, a 9-atom fragment (7-azaindole) is found in at least six clinical compounds targeting multiple kinases developed by different groups of researchers^14^. Medicinal chemists are making increasing use of fragments published by others to enable internal drug discovery efforts, in some cases leading to clinical compounds^15,16^. The dozens of fragment structures reported here can inspire myriad ideas well-suited to a collaborative approach, and indeed a consortium of researchers called the COVID Moonshot has been accepting public suggestions for molecules. These are being computationally triaged, synthesized, and tested, with experimental data made publicly available.

## The path ahead

There is still much to do. To paraphrase Winston Churchill, this is probably not even the end of the beginning. In the case of the first FBDD-derived drug approved, vemurafenib, it took just under a year to go from fragment to clinical candidate, an extraordinarily short amount of time for small molecule drug discovery. Functional activities for the fragments are just starting to be reported, and while some have sub-micromolar IC_50_s, they will likely need to be improved by orders of magnitude. Cell permeability, selectivity, pharmacokinetics, pharmacodynamics, and toxicity of improved molecules all remain to be assessed and optimized. Even if successful, it could be years before any candidates stemming from this work enter the clinic.

Although vaccines or antibody-based treatments may well be available before a small molecule is approved, the work on M^pro^ is still worth pursuing. Vaccines may not be 100% effective in preventing infection, so drugs to treat COVID-19 will still be needed. SARS-CoV-2 is the third coronavirus to afflict us since the beginning of the century, and there is no reason to think we will not see more. While SARS seems to have disappeared, there are still no approved treatments for MERS, which has a higher mortality rate but a lower transmission rate. Given the close conservation of M^pro^, fragment-derived inhibitors could be broadly active against such threats. The fragments discovered by Douangamath et al.—along with efforts carried out worldwide—represent the first steps in a long and uncertain journey. But perhaps someday, one of them will be recognized as a giant leap for humankind.
